# Special Issue “Alzheimer’s Disease—115 Years after Its Discovery”

**DOI:** 10.3390/biomedicines12030478

**Published:** 2024-02-21

**Authors:** Susana Cardoso, Cristina Carvalho, Sónia C. Correia

**Affiliations:** 1 CNC—Center for Neuroscience and Cell Biology, University of Coimbra, 3004-504 Coimbra, Portugal; cristina.im.carvalho@gmail.com (C.C.); sonia.s.correia84@gmail.com (S.C.C.); 2 CIBB—Center for Innovative Biomedicine and Biotechnology, University of Coimbra, 3004-504 Coimbra, Portugal; 3 Institute for Interdisciplinary Research, University of Coimbra, 3030-789 Coimbra, Portugal

Alzheimer’s disease (AD) is a progressive and multifactorial disease that significantly compromises the lives of millions of people worldwide. Since its first description more than 115 years ago, countless investigational efforts have been conducted in the AD field in order to improve the quality of life of patients affected by the disorder. The present Special Issue (SI), entitled “Alzheimer’s Disease—115 Years after Its Discovery”, gathers 14 original works, 11 review manuscripts and 1 meta-analysis that together chart the journey of AD and offer the hope of discovering new opportunities to prevent or arrest AD progression.

Despite a worldwide effort, AD is not a fully characterized disease, mainly due to its multitude of genetic and environmental risk factors that combine into several pathophysiological mechanisms. As a result, disease-modifying therapies are limited, and those that are available only provide a marginal benefit to AD patients [1]. With this in mind, García-Morales and colleagues [2] elaborated a comprehensive narrative review updating current knowledge on the pathophysiological mechanisms of AD, diagnostic methods and the therapeutic approach to AD. Exploring the mechanisms behind AD development and identifying reliable blood-based biomarkers could also revolutionize AD diagnosis and treatment. In a meticulously crafted review, Mankhong and colleagues [3] traced the evolution of AD biomarkers from the past to the present day. Additionally, in this SI, Phan and Cho [4] present a new technique intended to improve the field of serum biomarkers and enable the early diagnosis of AD. These authors developed new aptamer-mediated biosensors that enable the rapid monitoring of plasma phosphorylated tau (p-tau) at residue threonine 231, which is an early p-tau isotope often used for the diagnosis of AD. In a complementary way, Ma and colleagues [5] present a systematic review and meta-analysis strengthening the concept that the Aβ_42_/p-tau ratio is the most robust indicator for predicting the transition from mild cognitive impairment (MCI) to AD. To supplement these advances, a recent study developed a novel tau-based biomarker that allows one to distinguish AD from other neurodegenerative pathologies [6]. With the rationale that plasma tau proteins can originate from peripheral sources as well as the brain, these authors developed techniques to specifically detect blood levels of brain-derived tau that would reliably reflect cerebrospinal fluid (CSF) total tau [6]. From a different perspective, Jullienne and colleagues [7] provide a comprehensive overview of the human magnetic resonance imaging (MRI) and positron emission tomography (PET) imaging landscape, setting the stage for a detailed exploration of preclinical imaging in two of the most popular AD mouse models, the 3xTg-AD and the 5xFAD models. Balaji and collaborators [8] also proposed a new multimodal deep learning approach for the early detection of AD (Multi-DL) through the development of a hybrid model that can identify/predict the conversion of stable MCI to AD using MRI and PET data and standard neuropsychological test scores. Despite limitations, this exciting study also suggests that deep neural networks may be trained to automatically discover imaging biomarkers indicative of AD, calling attention to the potential advantages of this new era of biomedical engineering techniques. The use of neuroimaging techniques like multimodal electroencephalography (EEG) and functional near-infrared spectroscopy (fNIRS) was also shown to be a valuable approach to support early AD diagnosis [9]. As Chiarelli and co-workers [10] nicely demonstrated, these neuroimaging approaches allow one to evaluate the neurovascular coupling of an individual and infer alterations to cerebral blood flow and oxygen delivered to the brain. Notably, the authors state that this procedure can be carried out in ambulatory settings and easily used by clinicians.

Over the past few decades, science and medicine have witnessed a paradigm shift from curative to preventive strategies, embracing a personalized approach to clinical interventions tailored for specific patients and situations. Sex-related differences represent an emerging factor and example for the need to develop and consider AD patient stratification and personalized treatment. As documented in current research, women are more susceptible and present more aggressive clinical manifestations of the disease than men [11], a paradigm that could be due to genetics, according to a recent study conducted using artificial intelligence (AI), namely a machine learning (ML) approach [12]. Notably, those findings not only increase knowledge about sex-specific mechanisms that contribute to AD but also highlight the importance of tailored therapeutic approaches and the potential contribution of AI to achieving such outcomes [12]. To contribute to this field, Silva-Spínola and colleagues [13] provide a compelling and detailed discussion of the main characteristics, current applicability and future perspectives of the implementation of AI in healthcare, particularly in AD. As further support, in this SI, Rao and co-workers [14] present a paper that highlights the positive impact of a personalized therapeutic program (ReCODE) to lessen AD risk factors and improve and preserve cognitive function, thus paving the way for a new perspective on AD treatment and calling for larger controlled studies to provide further validation.

Memory decline is one of the major features of AD, with memory consolidation being an important mechanism in retaining new memories over time. Consequently, delving into the molecular foundations of memory consolidation has promise for making substantial strides in forthcoming therapeutic strategies for AD. One possible candidate is the activity-regulated cytoskeletal protein (ARC), a neuron-specific, post-synaptic protein that was found to be decreased in AD experimental models [15]. Corroborating data were obtained in a work by Leung and colleagues [16], clearly highlighting that ARC is a master regulator of neuronal-activity-dependent gene expression that plays a significant role in the pathophysiology of AD.

Neuronal dysfunction and death are prominent features of AD pathogenesis related to changes of an unknown origin in the APP metabolism and Aβ overproduction. To gain further insights into the molecular basis of Aβ toxicity in AD, Caballero and colleagues [17] highlighted the importance of better understanding how the primary Ca^2+^ entry pathway can be activated by Aβ oligomers in rat hippocampal and cerebellar neurons, leading to mitochondrial Ca^2+^ overload, with consequent neuronal death via apoptosis. Additionally, in a search for the mechanistic roots underlying aberrant Aβ accumulation in the AD brain, a role was identified for the water channel aquaporin 5 (AQP5), which is particularly abundant in the salivary glands, during the progression of the disease [18]. Particularly, the authors found impaired AQP5 expression in the submandibular glands in APP/PS1 mice and AD individuals, whereas increased AQP5 expression was detected in the APP/PS1 cerebral cortex. It is known that in the brain, AQPs are responsible for mediating the transport of water between different fluid compartments, including the CSF, contributing to its production and homeostasis [19].AD research also discloses the involvement of AQPs in the removal of waste products, such as soluble Aβ in the CSF [20]. More recently, a link between failure in Aβ clearance and alterations in the glymphatic system in which AQPs participate has been suggested to occur in aged and AD brains [21]. To deepen the understanding of this subject, Gião and colleagues [22] also provide a comprehensive overview of the current understanding of the roles of the choroid plexus (CP) and blood–cerebrospinal fluid barrier (BCSFB) in AD pathophysiology. Particularly, these authors discussed how changes in CSF secretion and dynamics, inflammation, oxidative stress and BCSFB integrity and transport can hinder the clearance of the Aβ peptide, leading to its accumulation and worsening pathology.

Growing evidence suggests that AD and cardiovascular disease (CVD) are interconnected pathologies sharing several mechanisms of disease [23]. In order to deepen these mechanistic pathways and identify possible common disease-associated signatures, Lee and colleagues [24] performed a blood genome-wide transcriptome analysis, having identified two common upstream genes (GPBP1 and SETDB2) between AD and CVD in gene regulatory networks. In due course, this new knowledge may contribute to elucidating the shared mechanisms between these two diseases, as well as to implementing personalized preventive strategies [25]. In fact, according to recent findings, there is a significant association between midlife cardiovascular conditions and midlife cognitive decline that varies between men and women depending on the risk factors involved [26]. Also, taking advantage of the 3xTg-AD mouse model of AD, Jullienne and collaborators [27] observed age-related alterations in individual vessels and the vascular network of the cortex with modest gender-dependent differences. Notably, profound vascular remodeling at 4–6 months of age was also detected in both female and male mice, which concurs with the known transition to cognitive impairment and Aβ deposition in this mouse model.

Disturbances along the gut–brain axis, defined as a bidirectional communication network between the gastrointestinal tract and central nervous system, were recently recognized as “triggers” and/or “enhancers” of the neuroinflammatory events that occur in the AD brain [28]. It can be observed that the microbiome of AD individuals predominantly constitutes proinflammatory bacteria and has lower levels of bacteria that have the potential to synthesize butyrate, an anti-inflammatory bacterial metabolite [29]. Interestingly, data show that treating healthy animals with AD-diseased microbiota elicits AD-like symptoms [30], whilst the opposite, i.e., introducing healthy microbiota into an AD mouse model, lessens cognitive disabilities, neuroinflammation and AD hallmarks [31]; such findings emphasize the relevance of investigating microbiota–gut–brain axis-targeted interventions in AD [31]. Along this line, Marcos Pasero and colleagues [32] summarize here current knowledge about the efficacy of several non-pharmacological strategies and bioactive immunometabolic compounds in targeting critical innate immune signals and cells, being able to modulate immune and metabolic response within the gut–brain axis in the context of AD pathology. Beyond the “inflammatory scenario” of AD, it is widely recognized that neuron–glia communication and cooperation is an essential pillar to many vital functions in the brain and is of the utmost importance during brain development [33]. In this sense, Rudnitskaya and co-workers [34] were able to obtain new pieces of evidence demonstrating significant disturbances in glial cell (astrocytes and microglia) support in the hippocampus and prefrontal cortex of senescence-accelerated OXYS rats during the first postnatal week. As the authors propose, those earlier developmental events may translate and/or contribute to the neurodegenerative process that occurs late in life in OXYS rats. In addition, Muñoz Herrera and Zivkovic [35] also present an elegant review on the involvement of microglia and cholesterol handling in AD pathology. Based on the well-established link between the apolipoprotein E (APOE) genotype and increased AD risk [36], these authors discuss how a high-cholesterol environment, particularly in the context of defects in the ability to transport cholesterol, can regulate microglial phenotype and function and the potential therapeutic application of statins. In a complementary approach, in this SI, Hong and colleagues [37] focused on determining the influence of the APOE genotype on HDL function and size in the context of AD. Their findings revealed differing mechanisms of HDL deficiency in APOE4 carriers compared to non-carriers, irrespective of the presence of an AD diagnosis. Concurrent findings, however, exposed a positive association between the amounts of small HDL particles in the CSF of individuals aged ≥ 60 years and their cognitive performance that was independent of age, sex, education and APOE genotype [38]. Such disparities underscore the importance of conducting further studies to unravel how changes in HDL composition and structure contribute to AD pathology.

In the realm of potential therapies, a promising breakthrough in combating AD emerged from the work of Umeda and colleagues [39]. These authors demonstrated that the nasal administration of rifampicin inhibits the spread of tau oligomers and neurofibrillary tangle formation, preventing synapse and neuronal loss in the hippocampus, and maintained normal cognitive function in a newly developed tauopathy mouse model of AD, supporting its efficacy as a promising therapeutic tool in AD pathology. Also in this SI, Trobec and colleagues [40] studied the effects of different ruthenium(II) compounds for its anti-cholinesterase (ChE) and anti- glutathione S-transferase (GST) activities, a subject of interest for AD since these enzymes are known to be involved in AD pathology, and the data obtained can lay a foundation for further preclinical testing as ChE- and GST-inhibitory drugs. In addition to these possible therapeutics, Choudhury and colleagues [41] provide an overview of the close association between ceramides and the occurrence of AD-related pathological features and discuss the therapeutic benefits of targeting ceramide biosynthesis to “fight” this devastating neurodegenerative disease. Vicente-Zurdo and colleagues [42] also present here some novel rivastigmine derivatives with anti-AD properties, as shown by the inhibition of cholinesterase activity, Aβ self-aggregation and Cu(II)-induced aggregation, as well as reductions in neurotoxicity and oxidative damage triggered by the amyloidogenic peptide Aβ_42_ toxicity in a human neuronal in vitro model. Considering the recognized role of the vascular component in the onset and progression of AD pathology [43], in this SI, Grossmann [44] provides an overview of the potential therapeutic effects of targeting thrombin activity or production with direct oral anticoagulants (DOACs) to counteract cerebrovascular and neuronal dysfunction and consequently cognitive deterioration in individuals diagnosed with early-stage AD and with a low risk of major bleeding, highlighting the importance of drug repurposing. Lastly, Cho and colleagues [45] conducted a decade review analysis (from 2011 to 2021) of published articles on JNK inhibitors, focusing on those with a structural perspective and docking insights. Based on the knowledge that the JNK kinase is strongly linked with several pathological pathways of AD [46] and its activation is found to be triggered by increased Aβ levels [47], the authors gathered significant evidence for the potentiality of JNK inhibitors to overcome the disease. More recently, considerable pre-clinical studies have contributed to reinforcing the therapeutic potential of JNK3 inhibitors in AD [48,49].

AD research, similar to other fields of investigation, relies on the use of experimental models, either in vivo or in vitro, to decipher the disease’s pathological background and assess the usefulness of therapeutic approaches before possible clinical translation [50]. However, due to the diverse range of AD models available, each one with benefits and limitations, conflicting findings can occasionally occur. Over the past 25 years, the nematode *Caenorhabditis elegans* has gained significant attention in the AD field [51]. Using this AD model, Alvarez and colleagues [52] deeply explored existing data in this model, ranging from the fundamental molecular mechanisms of the disease to the search for new therapeutic targets. The capacity of this nematode to generate knock-out or overexpression models of any gene, single or combined, and to carry out toxicity, recovery or survival studies in short timeframes with many individuals and at a low cost is also highlighted in this review. These comprehensive surveys shed light on the relevance of need and for evaluating different experimental settings and models to overcome the complexity of AD pathogenesis [53].

We expect that this SI provides important insights to build hope for better prognosis, diagnosis and treatment of AD. We also would like to sincerely thank all the authors for their valuable contributions, all the scientists who kindly agreed to participate in peer reviews and the managing editor of this SI of *Biomedicines*.
